# Exposure to Agent Orange and Risk of Bladder Cancer Among US Veterans

**DOI:** 10.1001/jamanetworkopen.2023.20593

**Published:** 2023-06-27

**Authors:** Stephen B. Williams, Jessica L. Janes, Lauren E. Howard, Ruixin Yang, Amanda M. De Hoedt, Jacques G. Baillargeon, Yong-Fang Kuo, Douglas S. Tyler, Martha K. Terris, Stephen J. Freedland

**Affiliations:** 1 Division of Urology, Department of Surgery, The University of Texas Medical Branch at Galveston; 2 Urology Section, Department of Surgery, Durham Veterans Affairs Medical Center, Durham, North Carolina; 3 Duke Cancer Institute Biostatistics Shared Resource, Durham, North Carolina; 4 Division of Epidemiology, Department of Medicine, Sealy Center on Aging, The University of Texas Medical Branch at Galveston; 5 Department of Surgery, The University of Texas Medical Branch at Galveston; 6 Section of Urology, Augusta University, Augusta, Georgia; 7 Section of Urology, Charlie Norwood Veterans Affairs Medical Center, Augusta, Georgia; 8 Department of Urology, Cedars-Sinai Medical Center, Los Angeles, California

## Abstract

**Importance:**

To date, limited data exist regarding the association between Agent Orange and bladder cancer, and the Institute of Medicine concluded that the association between exposure to Agent Orange and bladder cancer outcomes is an area of needed research.

**Objective:**

To examine the association between bladder cancer risk and exposure to Agent Orange among male Vietnam veterans.

**Design, Setting, and Participants:**

This nationwide Veterans Affairs (VA) retrospective cohort study assesses the association between exposure to Agent Orange and bladder cancer risk among 2 517 926 male Vietnam veterans treated in the VA Health System nationwide from January 1, 2001, to December 31, 2019. Statistical analysis was performed from December 14, 2021, to May 3, 2023.

**Exposure:**

Agent Orange.

**Main Outcomes and Measures:**

Veterans exposed to Agent Orange were matched in a 1:3 ratio to unexposed veterans on age, race and ethnicity, military branch, and year of service entry. Risk of bladder cancer was measured by incidence. Aggressiveness of bladder cancer was measured by muscle-invasion status using natural language processing.

**Results:**

Among the 2 517 926 male veterans (median age at VA entry, 60.0 years [IQR, 56.0-64.0 years]) who met inclusion criteria, there were 629 907 veterans (25.0%) with Agent Orange exposure and 1 888 019 matched veterans (75.0%) without Agent Orange exposure. Agent Orange exposure was associated with a significantly increased risk of bladder cancer, although the association was very slight (hazard ratio [HR], 1.04; 95% CI, 1.02-1.06). When stratified by median age at VA entry, Agent Orange was not associated with bladder cancer risk among veterans older than the median age but was associated with increased bladder cancer risk among veterans younger than the median age (HR, 1.07; 95% CI, 1.04-1.10). Among veterans with a diagnosis of bladder cancer, Agent Orange was associated with lower odds of muscle-invasive bladder cancer (odds ratio [OR], 0.91; 95% CI, 0.85-0.98).

**Conclusions and Relevance:**

In this cohort study among male Vietnam veterans, there was a modestly increased risk of bladder cancer—but not aggressiveness of bladder cancer—among those exposed to Agent Orange. These findings suggest an association between Agent Orange exposure and bladder cancer, although the clinical relevance of this was unclear.

## Key Points

**Question:**

Is there an association between Agent Orange exposure and risk of bladder cancer and/or aggressiveness of bladder cancer?

**Findings:**

In this cohort study of more than 2.5 million male Vietnam veterans, there was a modestly increased risk of bladder cancer—but not aggressiveness of bladder cancer—among veterans exposed to Agent Orange. Exposure to Agent Orange was associated with a significantly increased risk of bladder cancer, although the association was very slight (hazard ratio, 1.04; 95% CI, 1.02-1.06).

**Meaning:**

These findings suggest an association between exposure to Agent Orange and bladder cancer, although the clinical relevance of this was unclear.

## Teaser

This cohort study of more than 2.5 million male US Vietnam veterans examines the association between bladder cancer risk and exposure to Agent Orange.

## Introduction

There will be an estimated 81 180 new cases of bladder cancer and 17 100 deaths from bladder cancer in the US in 2022.^1^ Although most patients with bladder cancer receive a diagnosis of non–muscle-invasive disease (clinical stage T1 or less),^2^ approximately 20% to 40% of patients either present with or later develop muscle-invasive disease.^3^

Although smoking is one of the strongest bladder cancer risk factors, other risk factors include older age, male sex, and exposure to carcinogens, such as aromatic amines, inorganic arsenic, and polycyclic aromatic hydrocarbons.^4,5^ However, many bladder cancer cases cannot be explained by the known risk factors, leading to a need to identify new risk factors. One factor recently implicated is Agent Orange. To our knowledge, only limited data exist regarding the association of Agent Orange with bladder cancer risk and mortality, to date.^6,7,8,9,10,11,12,13^ The Institute of Medicine concluded that the association between Agent Orange exposure and bladder cancer outcomes is an area of needed research.^6^ Based on this and the aforementioned studies, the US Department of Veterans Affairs (VA) recently designated bladder cancer as a cancer caused by Agent Orange exposure.^6,14^ To provide a true measure of aggressive bladder cancer, it is crucial to study not just the diagnosis of bladder cancer but the aggressiveness of disease when diagnosed. However, to our knowledge, such a comprehensive analysis of Agent Orange and bladder cancer has not been done to date, mainly due to the small numbers of exposed cases, the lack of ability to identify Agent Orange exposure, and the lack of an ability to control for known confounders, particularly smoking, a major bladder cancer risk factor. To address these issues, we examined bladder cancer according to Agent Orange exposure using data from the largest integrated health system in the country, the VA health system.

## Methods

### Data Source

Using inpatient and outpatient data and fee-based or community care claims (ie, care provided outside the VA system for which the VA paid), we queried data from the VA Informatics and Computing Infrastructure (VINCI) to identify all veterans seen at any VA health system site from January 1, 2001, to December 31, 2019. Key to this proposal is the fact that all data from all VA sites are integrated into a common electronic medical record collated in VINCI. This study was approved by the institutional review boards at the Durham VA Medical Center, The University of Texas Medical Branch, and the US Army Medical Research and Development Command Human Research Protection Office. This study was conducted using a waiver of informed consent because the study met the following criteria: (1) the use or disclosure of the protected health information involves no more than a minimal risk to the privacy of individuals, (2) the research could not practicably be conducted without the waiver or alteration, and (3) the research could not practicably be conducted without access to and use of the protected health information. This study followed the Strengthening the Reporting of Observational Studies in Epidemiology (STROBE) reporting guideline for cohort studies.^15^

### Study Cohort

A total of 25 144 713 veterans enrolled in the VA nationwide from 2001 to 2019 were identified. This cohort study included 3 520 249 veterans who met the following criteria: active users of the VA system (defined as ≥2 medical office visits within 5 years); served in the Vietnam conflict; served in the military branch of Air Force, Army, Coast Guard, Navy, or Marine Corps; not transgender; 18 to 75 years of age at enlistment; and not deceased prior to January 1, 2001. To create a more homogeneous cohort that had opportunity for Agent Orange exposure, we then limited the study to men (as very few female veterans among those enrolled in the VA nationwide were exposed to Agent Orange) and excluded veterans missing pertinent data for matching, including race and ethnicity, date of birth, and military branch. After inclusions and exclusions were applied, the cohort consisted of 868 912 veterans exposed to Agent Orange and 2 427 677 controls. Next, we matched each veteran exposed to Agent Orange to 3 controls on factors associated with Agent Orange exposure using a stepwise algorithm, broadening the matching criteria at each stage. In the first step, we matched 67% of the controls to veterans exposed to Agent Orange on age at service entry (18-20, 21-22, or ≥23 years), military branch (Air Force, Army, Coast Guard, Navy, or Marine Corps), race and ethnicity (Black, White, or other [American Indian or Alaska Native, Asian, Native Hawaiian or Pacific Islander, and biracial]), and exact year of service entry. Subsequently, we matched 11% more controls to veterans exposed to Agent Orange following the same criteria but loosening year of service entry by ±1 year (1%), ±2 years (2%), ±3 years (4%), and ±4 years (4%) in iterative steps, for a total of 1 900 331 controls or 78% of the initial pool. In the end, 633 483 veterans exposed to Agent Orange (73%) were matched; among those matched, 99% had 3 matched controls and less than 1% had just 2 or 1 controls (Figure; eTable 1 and eTable 2 in Supplement 1).

**Figure.**
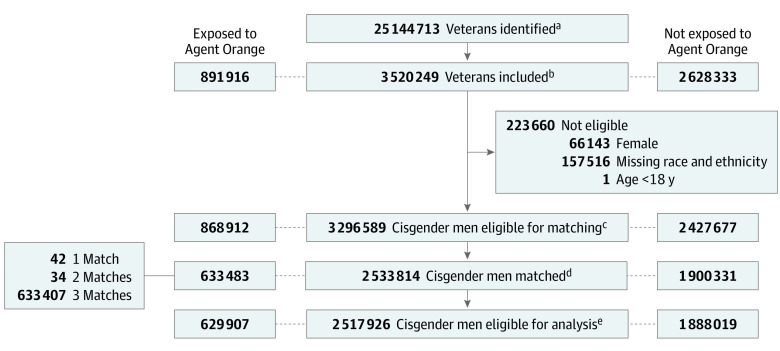
Diagram Illustrating Cohort Selection Process and Matching ^a^Identified from query of Veterans Affairs (VA) Health System entry date between 2001 and 2019. ^b^Included based on the following criteria: active users of the VA system; served in the Vietnam conflict; served in the military branch of Air Force, Army, Coast Guard, Navy, or Marine Corps; not transgender; age at enlistment 18 to 75 years; and not deceased prior to 2001. ^c^Men aged 18 years or older at service entry who were not missing data on race and ethnicity were eligible for matching. ^d^Matched on race and ethnicity, age groups at service entry, military branch, and year of service entry. For year of service entry, 86% of matched controls were matched on the exact year, and 14% were matched in subsequent steps using loosened criteria (±1-4 years). ^e^Patients were excluded if they were missing follow-up data or had a VA entry date the same as the most recent follow-up date.

### Exposure Data

The group exposed to Agent Orange consisted of veterans with documented exposure to Agent Orange–containing herbicide agents during active military duty. VINCI has an Agent Orange variable that designates exposure status as yes or no. Submitted documents fulfilling these requirements are then verified by a select VA committee.^16^ Date of military deployment to Vietnam was recorded as the index date for Agent Orange exposure. Patients in our study who had been exposed to Agent Orange were not defined as being exposed to Agent Orange because they also had a bladder cancer diagnosis; we used a landmark analysis in which we excluded bladder cancer diagnoses or patients who died or were lost to follow-up prior to 2001.

### Study Covariates

From VINCI, data captured for each patient included race and ethnicity (self-reported by patient or clinician) and other demographic characteristics (date of birth, sex, height, and weight), military service details (date of service entry, military branch, and eras served), and medical comorbidities (including date of onset, history of Agent Orange exposure, smoking history, and date of death [if applicable]). Visits with VA clinicians were captured and used to ensure the patient met our definition of a regular VA user. Smoking history was categorized as never smoker, former smoker, or current smoker. We also captured patients’ clinical and socioeconomic status information. Age was calculated at the date of VA entry (patient’s first visit in the VA system after January 1, 2001). Body mass index (BMI) was calculated as weight in kilograms divided by height in meters squared using each patient’s median height in the medical record and the weight closest to VA entry, after removing extreme values. Socioeconomic status was defined as the percentage living above the federal poverty level by linking each patient’s zip code of primary residence to 2014 American Community Survey^17^ estimates of the poverty rate and was categorized into quartiles. Comorbidities were assessed from claims data using the Klabunde modification of the Charlson Comorbidity Index (CCI) summed to the year after VA enrollment.^18^

### Natural Language Processing

Given the large cohort and the inability to capture disease stage information for all veterans with bladder cancer, along with the fact that the VA tumor registry lacks disease stage information for approximately two-thirds of veterans,^19^ we applied a natural language processing model that was previously developed, based on pathology reports to determine muscle invasion (yes or no) at the time of diagnosis, which had 94% accuracy in a validation set to separate veterans into groups according to whether they had muscle invasion (yes or no).^20^

### Outcomes

Bladder cancer incidence was defined as the total number of cases within the study period divided by the summed person-years and was multiplied by 1000 to get incidence per 1000 person-years. The primary outcome of interest was time to bladder cancer diagnosis. The secondary outcome was depth of invasion (muscle-invasive vs non–muscle-invasive bladder cancer) at the time of diagnosis among those with a diagnosis of bladder cancer.

### Statistical Analysis

Statistical analysis was performed from December 14, 2021, to May 3, 2023. Differences in demographic features by Agent Orange exposure were examined using the *t* test for normally distributed continuous variables, the Wilcoxon rank sum test for nonnormally distributed continuous variables, and the χ^2^ test for categorical variables. With a very large sample size, we had very high power to detect small differences as statistically significant. As such, standardized differences (*d*) were computed between Agent Orange groups, defined as the difference in mean values or proportions divided by the SE and interpreted as clinically relevant if *d* ≥ 0.10. Univariable and multivariable Cox proportional hazards regression models were used to test whether Agent Orange exposure was associated with risk of bladder cancer. Although we matched veterans on variables such as age at time of Vietnam service entry, or when they would have been exposed to Agent Orange, most data queried from VINCI (such as diagnoses and laboratory test results) did not become reliably available until the year 2000.^21^ This study thus used a landmark analysis whereby veterans were excluded if they received a diagnosis of bladder cancer, were deceased, or lost to follow-up prior to January 1, 2001. The first contact with the VA system starting in 2001 or after (VA entry) served as time zero in our models. Multivariable models were adjusted for age at VA entry, year of VA entry, race and ethnicity, BMI at VA entry, military branch, smoking status at VA entry, CCI, and socioeconomic status. Interactions were tested between Agent Orange exposure and year of service entry (tertials), smoking status (current, former, never, or unknown), age (younger than vs older than the median), BMI (<25, 25-29.9, ≥30, or unknown), and CCI (0, 1, 2, or ≥3) by including a cross-product term in the model. If no significant interactions were found, results were adjusted for these factors. If significant interactions were found, results were stratified by the effect modifier and reported in eTable 4 and eTable 5 in Supplement 1. Among veterans with a diagnosis of bladder cancer, we used univariable and multivariable logistic regression to test whether Agent Orange was associated with muscle invasiveness at diagnosis. Multivariable models were adjusted for the same covariates listed but with baseline variables at bladder cancer diagnosis as opposed to VA entry. All *P* values were from 2-sided tests and results were deemed statistically significant at *P* < .05, and all analyses were performed using SAS Enterprise Guide, version 8.2 software (SAS Institute Inc).

## Results

Among 2 517 926 male veterans (median age at VA entry, 60.0 years [IQR, 56.0-64.0 years]) who met inclusion criteria, there were 629 907 (25.0%) veterans with Agent Orange exposure and 1 888 019 (75.0%) matched veterans without Agent Orange exposure. (Table 1; Figure). There were fewer current smokers (20.9% vs 23.7%; *P* < .001) and fewer comorbidities (CCI score of ≥3: 17.3% vs 18.7%; *P* < .001) among veterans exposed to Agent Orange vs unexposed veterans. Although several variables were statistically different between veterans exposed to Agent Orange and unexposed veterans at baseline, the differences were small and did not meet our criterion for clinically meaningful differences.

**Table 1.** Male Patient Characteristics by Agent Orange Exposure

Characteristics	Not exposed to Agent Orange (n = 1 888 019)	Exposed to Agent Orange (n = 629 907)	*d* ^a^
Age at service entry, y^b^			
<21	1 288 214 (68.2)	429 822 (68.2)	0.00
21-22	328 329 (17.4)	109 500 (17.4)	
≥23	271 476 (14.4)	90 585 (14.4)	
Year of service entry^b^			
≤1965	644 058 (34.1)	202 602 (32.2)	0.08
1966-1968	630 970 (33.4)	236 045 (37.5)	
1969-1975	612 991 (32.5)	191 260 (30.4)	
Race and ethnicity^b^			
Black	250 297 (13.3)	83 559 (13.3)	0.00
White	1 573 323 (83.3)	524 859 (83.3)	
Other^c^	643 99 (3.4)	21 489 (3.4)	
Branch of service^b^			
Army	1 066 306 (56.5)	355 803 (56.5)	0.00
Air Force	284 572 (15.1)	94 894 (15.1)	
Navy or Coast Guard	357 673 (18.9)	119 333 (18.9)	
Marine Corps	179 468 (9.5)	59 877 (9.5)	
Year of VA entry			
2001-2004	869 767 (46.1)	233 387 (37.1)	0.19
2005-2009	504 631 (26.7)	197 327 (31.3)	
2010-2014	338 601 (17.9)	137 974 (21.9)	
2015-2019	175 020 (9.3)	61 219 (9.7)	
Age at VA entry, y			
Mean (SD)	60.3 (6.7)	61.1 (6.6)	0.11
Median (IQR)	60.0 (55.0-64.0)	60.0 (56.0-65.0)	
BMI at VA entry			
<25	368 599 (19.5)	105 131 (16.7)	0.09
25-29.9	637 853 (33.8)	217 719 (34.6)	
≥30	683 726 (36.2)	243 301 (38.6)	
Unknown	197 841 (10.5)	63 756 (10.1)	
Smoking status at VA entry			
Current smoker	447 564 (23.7)	131 876 (20.9)	0.09
Former smoker	695 354 (36.8)	249 994 (39.7)	
Never smoked	472 556 (25.0)	163 348 (25.9)	
Unknown	272 545 (14.4)	84 689 (13.4)	
CCI score within 1 y after VA entry			
0	859 614 (45.5)	296 821 (47.1)	0.03
1	445 220 (23.6)	147 518 (23.4)	
2	229 798 (12.2)	76 320 (12.1)	
≥3	353 387 (18.7)	109 248 (17.3)	
% Living above the poverty line			
Missing	71 469	22 844	0.09
Median (IQR)	84.8 (78.3-90.2)	85.5 (79.2-90.7)	

Abbreviations: BMI, body mass index (calculated as weight in kilograms divided by height in meters squared); CCI, Charlson Comorbidity Index; VA, Veterans Affairs.

^a^ The standardized difference or effect size.

^b^ Matching variables.

^c^ Included American Indian or Alaska Native, Asian, Native Hawaiian or Pacific Islander, and biracial individuals.

During a follow-up of 28 672 655 person-years, there was a total of 50 781 bladder cancer diagnoses (2.0%) (2.1% vs 2.0% among those exposed to Agent Orange vs those unexposed) recorded, for an overall bladder cancer incidence of 1.77 cases per 1000 person-years (eTable 3 in Supplement 1). With a follow-up of 7 292 654 and 21 380 001 person-years among veterans exposed to Agent Orange vs those unexposed, respectively, the incidence of bladder cancer was 1.84 vs 1.75 cases per 1000 person-years, respectively.

Among all veterans, after multivariable adjustment, there was an increased risk of bladder cancer (hazard ratio [HR], 1.04; 95% CI, 1.02-1.06) among those with Agent Orange exposure (Table 2). There was a decreased risk of bladder cancer among veterans of other racial groups (HR, 0.76; 95% CI, 0.72-0.80) and Black veterans (HR, 0.65; 95% CI, 0.63-0.67) vs White veterans. Marine Corps veterans had the greatest risk of bladder cancer (HR, 1.07; 95% CI, 1.04-1.10) compared with Army veterans. There was an increased risk of bladder cancer among current smokers vs never smokers (HR, 2.02; 95% CI, 1.97-2.07) and among those with increased comorbidities (CCI ≥3 vs 0: HR, 2.21; 95% CI, 2.16-2.26). Veterans in the highest socioeconomic quartile and least likely to live in poverty were most likely to receive a diagnosis of bladder cancer (HR, 1.07; 95% CI, 1.05-1.10).

**Table 2.** Univariable and Multivariable Associations Between Agent Orange Exposure and Bladder Cancer Diagnosis (N = 2 517 926)

Variable	Hazard ratio (95% CI)	*P* value
Univariable		
Agent Orange exposure		
No	1 [Reference]	<.001
Yes	1.06 (1.03-1.08)	
Multivariable		
Agent Orange exposure		
No	1 [Reference]	<.001
Yes	1.04 (1.02-1.06)	
BMI at VA entry		
<25	1 [Reference]	<.001
25-29.9	0.94 (0.92-0.96)	
≥30	0.91 (0.89-0.93)	
Unknown	0.90 (0.87-0.94)	
Age at VA entry	1.04 (1.04-1.04)	<.001
Year of VA entry		
2001-2004	1 [Reference]	<.001
2005-2009	1.10 (1.07-1.12)	
2010-2014	1.29 (1.25-1.33)	
2015-2019	1.49 (1.42-1.57)	
Race and ethnicity		
White	1 [Reference]	<.001
Black	0.65 (0.63-0.67)	
Other^a^	0.76 (0.72-0.80)	
Branch of service		
Army	1 [Reference]	<.001
Air Force	0.96 (0.93-0.98)	
Marine Corps	1.07 (1.04-1.10)	
Navy or Coast Guard	1.00 (0.98-1.03)	
Year of service entry		
≤1965	1 [Reference]	<.001
1966-1968	0.95 (0.93-0.97)	
1969-1975	0.88 (0.86-0.91)	
Smoking status at VA entry		
Never smoker	1 [Reference]	<.001
Current smoker	2.02 (1.97-2.07)	
Former smoker	1.41 (1.37-1.44)	
Unknown	1.43 (1.38-1.48)	
CCI at VA entry		
0	1 [Reference]	<.001
1	1.13 (1.11-1.16)	
2	1.85 (1.80-1.90)	
≥3	2.21 (2.16-2.26)	
% Living above poverty line		
0-78.6	1 [Reference]	<.001
78.7-85.1	1.02 (0.99-1.05)	
85.2-90.4	1.05 (1.03-1.08)	
90.5-100	1.07 (1.05-1.10)	
Unknown zip code	0.98 (0.93-1.03)	

Abbreviations: BMI, body mass index (calculated as weight in kilograms divided by height in meters squared); CCI, Charlson Comorbidity Index; VA, Veterans Affairs.

^a^ Included American Indian or Alaska Native, Asian, Native Hawaiian or Pacific Islander, and biracial individuals.

Further analyses were performed to evaluate whether interactions were present between Agent Orange exposure and age, year of service entry, BMI, smoking status, and CCI for estimating bladder cancer risk (Table 3). There was no significant interaction according to smoking, BMI, and CCI. Agent Orange exposure was associated with an increased risk of bladder cancer for veterans younger than the median age (HR, 1.07; 95% CI, 1.04-1.10), but not for veterans older than the median age (HR, 1.03; 95% CI, 0.99-1.05; *P* = .04 for interaction). Agent Orange exposure was associated with an increased risk for bladder cancer for veterans who entered the military between the years 1969 and 1975 (HR, 1.08; 95% CI, 1.04-1.12), but not for the earlier 2 tertials (*P* = .04 for interaction). Given significant interactions between median age and Agent Orange exposure and between year of service entry and Agent Orange exposure, we conducted separate analyses for both age groups (eTable 4 in Supplement 1) and the year of service entry groups (eTable 5 in Supplement 1).

**Table 3.** Hazard Ratios for Agent Orange Exposure and Time to Bladder Cancer Diagnosis Stratified by Median Age, BMI Group, Smoking Status, and CCI at VA Entry

Variable	Hazard ratio (95% CI)^a^	*P* value for interaction^b^
Age		
Above median	1.03 (0.99-1.05)	.04
Below median	1.07 (1.04-1.10)	
Year of service entry		
≤1965	1.02 (0.98-1.05)	.04
1966-1968	1.03 (0.99-1.06)	
1969-1975	1.08 (1.04-1.12)	
BMI		
<25	1.05 (1.00-1.10)	.46
25-29.9	1.04 (1.01-1.08)	
≥30	1.02 (0.99-1.05)	
Unknown	1.08 (1.00-1.16)	
Smoking status		
Never smoked	0.99 (0.94-1.03)	.07
Current smoker	1.06 (1.02-1.10)	
Former smoker	1.03 (1.00-1.07)	
Unknown	1.07 (1.01-1.13)	
CCI at VA entry		
0	1.04 (1.00-1.07)	.72
1	1.04 (1.00-1.09)	
2	1.04 (0.99-1.09)	
≥3	1.01 (0.97-1.05)	

Abbreviations: BMI, body mass index (calculated as weight in kilograms divided by height in meters squared); CCI, Charlson Comorbidity Index; VA, Veterans Affairs.

^a^ Hazard ratio for Agent Orange exposure (yes) among given group estimated from multivariable model with interaction term.

^b^ Corresponds to *P* value from interaction term between Agent Orange exposure and the given variable in multivariable model adjusted for the covariates in Table 2.

The association between Agent Orange exposure and muscle-invasive bladder cancer at diagnosis among those with a diagnosis of bladder cancer is shown in Table 4. There was a decreased odds of diagnosis of muscle-invasive bladder cancer among those with Agent Orange exposure (odds ratio [OR], 0.91; 95% CI, 0.85-0.98). We observed increased odds of muscle-invasive bladder cancer among Black vs White veterans (OR, 1.16; 95% CI, 1.05-1.28). Veterans in the highest socioeconomic quartile and least likely to live in poverty had the lowest odds of receiving a diagnosis of muscle-invasive bladder cancer (OR, 0.88; 95% CI, 0.80-0.96). Current smokers (OR, 1.10; 95% CI, 1.00-1.21) and former smokers (OR, 1.08; 95% CI, 1.00-1.18) had higher odds of receiving a diagnosis of muscle-invasive bladder cancer compared with veterans who never smoked. Age at bladder cancer diagnosis, year of bladder cancer diagnosis, and branch of service were not associated with diagnosis of muscle-invasive bladder cancer. No significant interactions were observed between Agent Orange exposure and age, BMI, smoking, or CCI for estimating muscle-invasion status (eTable 6 in Supplement 1).

**Table 4.** Univariable and Multivariable Associations Between Agent Orange Exposure and Muscle-Invasive Bladder Cancer Assessed From Diagnostic Transurethral Resection of Bladder Tumor Among Men With Bladder Cancer Diagnosis

Variable	Odds ratio (95% CI)	*P* value
Univariable		
Agent Orange exposure		
No	1 [Reference]	.004
Yes	0.90 (0.84-0.97)	
Multivariable		
Agent Orange exposure		
No	1 [Reference]	.009
Yes	0.91 (0.85-0.98)	
Age at bladder cancer diagnosis	1.00 (0.99-1.01)	.79
Year of bladder cancer diagnosis	1.00 (0.99-1.01)	.51
Race and ethnicity		
White	1 [Reference]	.003
Black	1.16 (1.05-1.28)	
Other^a^	0.87 (0.72-1.04)	
BMI at bladder cancer diagnosis		
<25	1 [Reference]	<.001
25-29.9	0.84 (0.78-0.90)	
≥30	0.77 (0.71-0.83)	
Unknown	0.55 (0.24-1.26)	
Branch of service		
Army	1 [Reference]	.35
Air Force	0.95 (0.86-1.04)	
Marine Corps	1.01 (0.92-1.12)	
Navy or Coast Guard	0.94 (0.87-1.02)	
Smoking status at bladder cancer diagnosis		
Never smoked	1 [Reference]	.04
Current smoker	1.10 (1.00-1.21)	
Former smoker	1.08 (1.00-1.18)	
Unknown	1.17 (1.05-1.31)	
CCI at bladder cancer diagnosis		
0	1 [Reference]	<.001
1	0.81 (0.73-0.90)	
2	1.00 (0.90-1.12)	
≥3	1.03 (0.94-1.12)	
% Living above the poverty line		
0-78.6	1 [Reference]	.006
78.7-85.1	1.02 (0.94-1.12)	
85.2-90.4	1.01 (0.92-1.10)	
90.5-100	0.88 (0.80-0.96)	
Unknown zip code	1.03 (0.88-1.21)	

Abbreviations: BMI, body mass index (calculated as weight in kilograms divided by height in meters squared); CCI, Charlson Comorbidity Index.

^a^ Included American Indian or Alaska Native, Asian, Native Hawaiian or Pacific Islander, and biracial individuals.

## Discussion

In 2014, the committee to review the health effects in Vietnam veterans of exposure to herbicides (Tenth Biennial Update) upgraded Agent Orange and bladder cancer risk from “no association” to “limited evidence.”^6^ Herein, we developed the largest nationwide cohort ever assembled to address bladder cancer risk and aggressiveness of bladder cancer according to Agent Orange exposure.

Our study revealed several important findings. First, we found that Agent Orange was associated with a 4% increased risk of bladder cancer and that this association depended on age and year of service entry. We found that Agent Orange was associated with a 7% increased risk of bladder cancer for veterans younger than the median age at VA entry, but no significant association was seen for veterans older than the median age. Thus, younger Vietnam veterans exposed to Agent Orange with potentially more life-years to develop bladder cancer were at greatest risk. This finding is critical because bladder cancer is more commonly diagnosed in older individuals, corresponding to the age of most Vietnam veterans who may have been exposed to Agent Orange.^6^ Given that the VA has recently designated bladder cancer as a cancer caused by Agent Orange exposure, veterans who were exposed to Agent Orange during military service may be eligible for a variety of VA benefits, including disability compensation for diseases associated with Agent Orange exposure. These benefits may extend to dependents and survivors of exposed individuals.^6,14^ Targeted screening could be implemented among people exposed to Agent Orange to detect bladder cancer earlier when it is more treatable. These findings persisted after controlling for numerous confounders using data from the largest equal-access system in the US and 28 672 655 person-years of follow-up.

Second, in the present study, other risk factors for bladder cancer were also tested, including race and ethnicity and smoking. It is well known that Black individuals have up to a 50% decreased incidence of bladder cancer than White individuals.^22,23,24,25^ Consistent with this statistic, we found a 35% decreased incidence of bladder cancer among Black veterans when controlling for other important confounders, including age and smoking status. Moreover, current smokers had up to a 2-fold increased risk of bladder cancer compared with never smokers, in accordance with prior findings.^4,5,26^ The fact that our findings mirror well-known population data for established bladder cancer risk factors lends credibility to our results.

Third, we attempted to control for social determinants of health, including poverty and access to care. The present findings were derived from the largest equal-access health system in the US. This is a key point regarding Agent Orange and bladder cancer risk because prior studies found either no or increased risk of bladder cancer among those with Agent Orange exposure.^9,10,11^ However, those studies were limited by either small numbers of patients, inadequate follow-up, and/or lack of controlling for important confounders (ie, smoking). Our study included more than 2 million matched veterans with long-term follow-up and adjusted for important confounders (ie, age, smoking, and BMI) in an equal-access setting to assess whether there is an association between Agent Orange and bladder cancer. Moreover, we found increased risk of bladder cancer among veterans who were least likely to live in poverty, suggesting that improved access to care and other improved social determinants of health may result in improved screening and, thus, detection of bladder cancer. This possibility is further supported by our findings noting decreased risk of muscle-invasive bladder cancer among those least likely to live in poverty, which would support the fact that these patients had earlier detection.

Fourth, we found decreased odds of muscle-invasive disease among veterans with Agent Orange exposure. Although we cannot determine causality given the retrospective nature of our study design, this observation may be due to earlier bladder cancer detection in the group exposed to Agent Orange. Moreover, given the increasing number of Agent Orange–related diseases, routine urinalysis and subsequent workup and/or screening may be initiated and may lead to earlier detection and less-advanced bladder cancer disease. We were unable to control for a possible dose-response relationship regarding Agent Orange exposure; prior studies have shown that an increased risk of bladder cancer aggressiveness may be associated with a higher concentration of and/or a close proximity to toxin exposure.^27,28^ Thus, the latency period (ie, Vietnam era) and weak dose-response relationship may be associated with our observed decreased likelihood of aggressive bladder cancer among veterans exposed to Agent Orange. Despite being in an equal-access setting, Black veterans had increased odds of muscle-invasive bladder cancer at diagnosis compared with White veterans. These findings mirror prior studies noting that Black vs White individuals have a decreased incidence of bladder cancer but more aggressive disease when diagnosed.^4,5,26^ Race-based differences in bladder cancer biology have been previously described wherein Black individuals may have more advanced disease at diagnosis compared with White individuals.^29^ Further investigation into bladder cancer incidence according to race and ethnicity are needed to better understand the interplay between biology, social determinants, and association with bladder cancer incidence and disease severity.^30,31^

### Limitations

This study has some limitations, and our findings must be considered within the context of the study design. First, veterans enrolled in the present study were male, with most having no comorbidities at the time of VA entry. Due to the limited numbers of women enrolled in the VA and exposed to Agent Orange during the Vietnam conflict, we excluded women to create a more homogeneous cohort. Given that most veterans with bladder cancer are diagnosed in their sixth decade of life or older (ie, the current age of most Vietnam veterans), our findings may not be generalizable to younger veterans. Second, while we performed a match design to control for several confounders, we cannot exclude potential selection bias and misclassification bias (ie, Agent Orange exposure due to lack of Agent Orange dosing or lack of exposure information, or CCI classification at VA entry) when using large population-based data. We used prior accepted methods and Agent Orange designation as defined by the VA.^27^ Moreover, we used year of service entry and year of VA entry variables to control for latency between Agent Orange exposure and bladder cancer diagnosis. We understand the inherent limitations in that there may have been a cohort of men with more-aggressive bladder cancer who died prior to 2001, thus confounding the study outcome. A further biological assessment of Agent Orange exposure and bladder cancer risk may support our findings.^32,33^ Third, while we attempted to control for the inherent selection bias and other confounders in this retrospective study, we acknowledge the limitations in using such a study design, including but not limited to unmeasured confounding.

## Conclusions

In a large population-based cohort, we found a slight increased risk of bladder cancer among veterans exposed to Agent Orange. In contrast, we found that Agent Orange exposure was associated with a decreased risk of muscle-invasive bladder cancer. These results support prior investigations and further support bladder cancer to be designated as an Agent Orange–associated disease.

## Notes

Supplement 1. **eTable 1.** Patients Characteristics by Agent Orange Exposure Prior to Matching **eTable 2.** Patients Characteristics by Agent Orange Exposure After Matching **eTable 3.** Bladder Cancer Incidence Rates by Agent Orange Exposure (N=2,517,926) **eTable 4.** Multivariable Associations Between Agent Orange Exposure and Bladder Cancer by Median Age At VA Entry (N=2,517,926) **eTable 5.** Multivariable Associations Between Agent Orange Exposure and Bladder Cancer by Year of Service Entry (N=2,517,926) **eTable 6.** Odds Ratios for Agent Orange Exposure and Muscle-Invasive Bladder Cancer (MIBC) Stratified by Median Age, Body Mass Index (BMI) Group, Smoking Status, and Charlson Comorbidity Index (CCI) at Bladder Cancer Diagnosis

Supplement 2.
Data Sharing Statement
