# Supplementary material for: Exposure to Agent Orange and Risk of Bladder Cancer Among US Veterans
Source: JAMA Netw Open. 2023 Jun 27;6(6):e2320593. doi: 10.1001/jamanetworkopen.2023.20593 (PMC10300690; doi:10.1001/jamanetworkopen.2023.20593)
Supplement: Supplement 1. — eTable 1. Patients Characteristics by Agent Orange Exposure Prior to Matching eTable 2. Patients Characteristics by Agent Orange Exposure After Matching eTable 3. Bladder Cancer Incidence Rates by Agent Orange Exposure (N=2,517,926) eTable 4. Multivariable Associations Between Agent Orange Exposure and Bladder Cancer by Median Age At VA Entry (N=2,517,926) eTable 5. Multivariable Associations Between Agent Orange Exposure and Bladder Cancer by Year of Service Entry (N=2,517,926) eTable 6. Odds Ratios for Agent Orange Exposure and Muscle-Invasive Bladder Cancer (MIBC) Stratified by Median Age, Body Mass Index (BMI) Group, Smoking Status, and Charlson Comorbidity Index (CCI) at Bladder Cancer Diagnosis [file jamanetwopen-e2320593-s001.pdf]

Supplementary Online Content

Williams SB, Janes JL, Howard LE, et al. Exposure to Agent Orange and risk of bladder cancer among US veterans. *JAMA Netw Open.* 2023;6(6):e2320593. doi:10.1001/jamanetworkopen.2023.20593

eTable 1. Patients Characteristics by Agent Orange Exposure Prior to Matching
eTable 2. Patients Characteristics by Agent Orange Exposure After Matching
eTable 3. Bladder Cancer Incidence Rates by Agent Orange Exposure (N=2,517,926)
eTable 4. Multivariable Associations Between Agent Orange Exposure and Bladder Cancer by Median Age At VA Entry (N=2,517,926)
eTable 5. Multivariable Associations Between Agent Orange Exposure and Bladder Cancer by Year of Service Entry (N=2,517,926)
eTable 6. Odds Ratios for Agent Orange Exposure and Muscle-Invasive Bladder Cancer (MIBC) Stratified by Median Age, Body Mass Index (BMI) Group, Smoking Status, and Charlson Comorbidity Index (CCI) at Bladder Cancer Diagnosis

This supplementary material has been provided by the authors to give readers additional information about their work.

© 2023 Williams SB et al. *JAMA Network Open.*

eTable 1. Patients Characteristics by Agent Orange Exposure Prior to Matching

Characteristics	Not Exposed (N=2,427,677)	AO Exposed (N=868,912)	p value	d[†]
**Age at service entry**			<0.001	0.07
<21	1641977 (67.64%)	613977 (70.66%)		
21-22	412042 (16.97%)	142563 (16.41%)		
>=23	373658 (15.39%)	112372 (12.93%)		
**Year of service entry**			<0.001	0.10
Mean (SD)	1966.1 (5.4)	1966.6 (3.5)		
Median	1967.0	1967.0		
Q1, Q3	1963.0, 1970.0	1965.0, 1969.0		
**Race**			<0.001	0.14
Black	369921 (15.24%)	93593 (10.77%)		
White	1975469 (81.37%)	748095 (86.10%)		
Other[ǂ]	82287 (3.39%)	27224 (3.13%)		
**Branch of service**			<0.001	0.28
Army	1273559 (52.46%)	527329 (60.69%)		
Air Force	456567 (18.81%)	95578 (11.00%)		
Navy / Coast Guard	481865 (19.85%)	135556 (15.60%)		
Marine Corps	215686 (8.88%)	110449 (12.71%)		
**Year of VA entry**			<0.001	0.31
Missing	7	1		
Mean (SD)	2006.7 (5.1)	2008.2 (5.0)		
Median	2005.0	2008.0		
Q1, Q3	2002.0, 2010.0	2004.0, 2012.0		
**Age at VA entry**			<0.001	0.15
Missing	7	1		
Mean (SD)	60.8 (7.5)	61.8 (6.3)		
Median	60.0	62.0		
Q1, Q3	55.0, 65.0	57.0, 66.0		
**BMI at VA entry**			<0.001	0.11
<25	477374 (19.66%)	140243 (16.14%)		
25-29.9	822150 (33.87%)	303001 (34.87%)		
≥30	867756 (35.74%)	338142 (38.92%)		
Unknown	260397 (10.73%)	87526 (10.07%)		
**Smoking status at VA entry**			<0.001	0.13
Current Smoker	560872 (23.10%)	169304 (19.48%)		
Former Smoker	899737 (37.06%)	353650 (40.70%)		
Never Smoked	613405 (25.27%)	231565 (26.65%)		
Unknown	353663 (14.57%)	114393 (13.17%)		
**CCI score within 1 year after VA entry**			<0.001	0.08
0	1113011 (45.85%)	424216 (48.82%)		

© 2023 Williams SB et al. *JAMA Network Open.*

eTable 1. Patients Characteristics by Agent Orange Exposure Prior to Matching

Characteristics	Not Exposed (N=2,427,677)	AO Exposed (N=868,912)	p value	d[†]
1	570304 (23.49%)	200170 (23.04%)		
2	296588 (12.22%)	104132 (11.98%)		
3+	447774 (18.44%)	140394 (16.16%)		
**% living above the poverty line**			<0.001	0.15
Missing	90238	28873		
Mean (SD)	83.1 (9.9)	84.5 (9.0)		
Median	84.7	86.0		
Q1, Q3	78.2, 90.2	79.8, 91.1		

[†]d represents the standardized difference or effect size

[⁑]Other comprised of Asian, American Indian or Alaskan Native, Native Hawaiian or Pacific Islander, and biracial races

© 2023 Williams SB et al. *JAMA Network Open.*

eTable 2. Patients Characteristics by Agent Orange Exposure After Matching

**Characteristics**	**Not Exposed (N=1,900,331)**	**AO Exposed (N=633,483)**	**p value**	**d[†]**
**Age at service entry***			1.0	0.00
<21	1295720 (68.18%)	431966 (68.19%)		
21-22	330613 (17.40%)	110183 (17.39%)		
>=23	273998 (14.42%)	91334 (14.42%)		
**Year of service entry***			<0.001	0.00
Mean (SD)	1966.4 (4.3)	1966.4 (4.0)		
Median	1967.0	1967.0		
Q1, Q3	1964.0, 1969.0	1965.0, 1969.0		
**Race***			<0.001	0.00
Black	251667 (13.24%)	83899 (13.24%)		
White	1583933 (83.35%)	527998 (83.35%)		
Other[ǂ]	64731 (3.41%)	21586 (3.41%)		
**Branch of service***			1.0	0.00
Army	1073004 (56.46%)	357682 (56.46%)		
Air Force	286734 (15.09%)	95578 (15.09%)		
Navy / Coast Guard	360091 (18.95%)	120033 (18.95%)		
Marine Corps	180502 (9.50%)	60190 (9.50%)		
**Age at VA entry**			<0.001	0.11
Missing	7	1		
Mean (SD)	60.3 (6.7)	61.1 (6.7)		
Median	60.0	60.0		
Q1, Q3	55.0, 64.0	56.0, 65.0		
**Year of VA entry**			<0.001	0.15
Missing	7	1		
Mean (SD)	2006.4 (5.0)	2007.1 (4.9)		
Median	2005.0	2006.0		
Q1, Q3	2002.0, 2010.0	2003.0, 2011.0		
**BMI at VA entry**			<0.001	0.09
<25	371068 (19.53%)	105734 (16.69%)		
25-29.9	640768 (33.72%)	218657 (34.52%)		
≥30	686573 (36.13%)	244287 (38.56%)		
Unknown	201922 (10.63%)	64805 (10.23%)		
**Smoking status at VA entry**			<0.001	0.09
Current Smoker	449994 (23.68%)	132506 (20.92%)		
Former Smoker	698768 (36.77%)	251181 (39.65%)		
Never Smoked	474545 (24.97%)	163979 (25.89%)		
Unknown	277024 (14.58%)	85817 (13.55%)		
**CCI score within 1 year after VA entry**			<0.001	0.04
0	864228 (45.48%)	297987 (47.04%)		
1	446714 (23.51%)	147822 (23.33%)		

© 2023 Williams SB et al. *JAMA Network Open.*

eTable 2. Patients Characteristics by Agent Orange Exposure After Matching

Characteristics	Not Exposed (N=1,900,331)	AO Exposed (N=633,483)	p value	d[†]
2	232216 (12.22%)	77256 (12.20%)		
3+	357173 (18.80%)	110418 (17.43%)		
**% living above the poverty line**			<0.001	0.09
Missing	71984	23010		
Mean (SD)	83.1 (9.8)	83.9 (9.3)		
Median	84.8	85.5		
Q1, Q3	78.3, 90.2	79.2, 90.7		

*matching variables

[†]d represents the standardized difference or effect size

[‡]Other comprised of Asian, American Indian or Alaskan Native, Native Hawaiian or Pacific Islander, and biracial races

© 2023 Williams SB et al. *JAMA Network Open.*

eTable 3. Bladder Cancer Incidence Rates by Agent Orange Exposure (N=2,517,926)

Group	N	Total Cases	Person Years	Incidence per 1,000 person years
All Patients	2517926	50781	28672655	1.77
AO Exposed	629907	13391	7292654	1.84
Not Exposed	1888019	37390	21380001	1.75
AO Exposed: Below median age	284073	6526	4183462	1.56
Not exposed: Below median age	912325	18217	12655519	1.44
AO Exposed: Above median age	345834	6865	3109192	2.21
Not exposed: Above median age	975694	19173	8724482	2.20

© 2023 Williams SB et al. *JAMA Network Open.*

eTable 4. Multivariable Associations Between Agent Orange Exposure and Bladder Cancer by Median Age At VA Entry (N=2,517,926)

		Below median age			Above median age		
**Variable**	**Parameter**	**HR**	**95% CI**	**p-value**	**HR**	**95% CI**	**p-value**
Agent Orange				<0.001			0.018
	No		Ref.			Ref.	
	Yes	1.06	(1.03, 1.09)		1.03	(1.01, 1.06)	
BMI at VA entry				<0.001			<0.001
	<25		Ref.			Ref.	
	25-29.9	0.96	(0.92, 0.99)		0.92	(0.88, 0.95)	
	≥30	0.93	(0.89, 0.96)		0.86	(0.83, 0.89)	
	Unknown	0.93	(0.88, 0.99)		0.90	(0.85, 0.95)	
Year of VA entry				<0.001			<0.001
	2001-2004		Ref.			Ref.	
	2005-2009	1.29	(1.25, 1.33)		1.04	(1.01, 1.08)	
	2010-2014	1.67	(1.53, 1.82)		1.28	(1.24, 1.33)	
	2015-2019	--	--		1.69	(1.60, 1.78)	
Race				<0.001			<0.001
	White		Ref.			Ref.	
	Black	0.66	(0.64, 0.69)		0.63	(0.61, 0.66)	
	Other	0.81	(0.76, 0.87)		0.72	(0.67, 0.78)	
Branch of service				0.14			<0.001
	Army		Ref.			Ref.	
	Air Force	0.96	(0.92, 1.00)		0.99	(0.96, 1.03)	
	Marine Corps	1.00	(0.96, 1.04)		1.12	(1.08, 1.18)	
	Navy / Coast Guard	0.98	(0.94, 1.01)		1.00	(0.97, 1.04)	
Year of service entry				<0.001			<0.001
	≤1965		Ref.			Ref.	
	1966-1968	0.83	(0.80, 0.85)		0.89	(0.86, 0.92)	
	1969-1975	0.67	(0.65, 0.70)		0.84	(0.81, 0.87)	
Smoking status at VA entry				<0.001			<0.001
	Never Smoked		Ref.			Ref.	
	Current Smoker	1.95	(1.88, 2.02)		1.96	(1.89, 2.04)	
	Former Smoker	1.38	(1.33, 1.43)		1.42	(1.37, 1.46)	
	Unknown	1.45	(1.39, 1.52)		1.39	(1.33, 1.45)	
CCI score at VA entry				<0.001			<0.001
	0		Ref.			Ref.	
	1	1.12	(1.08, 1.16)		1.16	(1.12, 1.20)	
	2	1.70	(1.64, 1.77)		2.06	(1.99, 2.14)	
	3+	1.93	(1.86, 2.00)		2.57	(2.48, 2.65)	
% living above the poverty line				<0.001			<0.001
	0-78.6%		Ref.			Ref.	
	78.7-85.1%	1.02	(0.98, 1.06)		1.03	(0.99, 1.06)	
	85.2-90.4%	1.06	(1.03, 1.10)		1.05	(1.01, 1.09)	
	90.5-100%	1.07	(1.03, 1.11)		1.08	(1.05, 1.13)	
	Unknown zipcode	0.98	(0.91, 1.05)		0.98	(0.91, 1.05)	

© 2023 Williams SB et al. *JAMA Network Open.*

eTable 5. Multivariable Associations Between Agent Orange Exposure and Bladder Cancer by Year of Service Entry (N=2,517,926)

		**1934-1965**			**1966-1968**			**1969-1975**		
**Variable**	**Parameter**	**HR**	**95% CI**	**p-value**	**HR**	**95% CI**	**p-value**	**HR**	**95% CI**	**p-value**
Agent Orange				0.20			0.11			0.004
	No		Ref.			Ref.			Ref.	
	Yes	1.02	(0.99, 1.06)		1.03	(0.99, 1.06)		1.06	(1.02, 1.10)	
BMI at VA entry				<0.001			<0.001			<0.001
	<25		Ref.			Ref.			Ref.	
	25-29.9	0.95	(0.91, 0.99)		0.94	(0.90, 0.99)		0.92	(0.87, 0.96)	
	≥30	0.93	(0.89, 0.97)		0.90	(0.86, 0.94)		0.88	(0.84, 0.93)	
	Unknown	0.87	(0.81, 0.93)		0.91	(0.85, 0.97)		0.95	(0.88, 1.02)	
Age at VA entry		1.03	(1.03, 1.03)	<0.001	1.05	(1.04, 1.05)	<0.001	1.05	(1.05, 1.06)	<0.001
Year of VA entry				<0.001			<0.001			<0.001
	2001-2004		Ref.			Ref.			Ref.	
	2005-2009	1.09	(1.05, 1.13)		1.08	(1.04, 1.13)		1.09	(1.05, 1.15)	
	2010-2014	1.25	(1.19, 1.32)		1.24	(1.16, 1.31)		1.30	(1.23, 1.38)	
	2015-2019	1.52	(1.40, 1.64)		1.34	(1.21, 1.48)		1.47	(1.34, 1.62)	
Race				<0.001			<0.001			<0.001
	White		Ref.			Ref.			Ref.	
	Black	0.67	(0.64, 0.70)		0.66	(0.63, 0.69)		0.61	(0.58, 0.65)	
	Other	0.78	(0.73, 0.85)		0.77	(0.70, 0.84)		0.72	(0.65, 0.80)	
Branch of service				<0.001			0.08			<0.001
	Army		Ref.			Ref.			Ref.	
	Air Force	1.00	(0.96, 1.03)		0.95	(0.90, 0.99)		0.91	(0.86, 0.97)	
	Marine Corps	1.13	(1.07, 1.19)		1.02	(0.97, 1.07)		1.07	(1.01, 1.13)	
	Navy / Coast Guard	1.03	(0.99, 1.06)		0.99	(0.95, 1.04)		0.98	(0.93, 1.02)	
Smoking status at VA entry				<0.001			<0.001			<0.001
	Never Smoked		Ref.			Ref.			Ref.	
	Current Smoker	1.94	(1.86, 2.02)		2.15	(2.05, 2.24)		2.01	(1.91, 2.12)	
	Former Smoker	1.31	(1.26, 1.36)		1.47	(1.41, 1.53)		1.50	(1.42, 1.57)	
	Unknown	1.33	(1.27, 1.40)		1.49	(1.41, 1.58)		1.53	(1.43, 1.63)	
CCI score at VA entry				<0.001			<0.001			<0.001
	0		Ref.			Ref.			Ref.	
	1	1.14	(1.10, 1.19)		1.13	(1.08, 1.18)		1.13	(1.08, 1.18)	
	2	1.82	(1.75, 1.90)		1.84	(1.76, 1.92)		1.92	(1.82, 2.02)	
	3+	2.22	(2.14, 2.30)		2.10	(2.02, 2.19)		2.36	(2.25, 2.47)	
% living above the poverty line				0.004			<0.001			0.28
	0-78.6%		Ref.			Ref.			Ref.	
	78.7-85.1%	1.01	(0.97, 1.05)		1.06	(1.02, 1.11)		0.98	(0.93, 1.02)	
	85.2-90.4%	1.03	(0.99, 1.08)		1.11	(1.06, 1.15)		1.01	(0.96, 1.06)	
	90.5-100%	1.07	(1.03, 1.12)		1.11	(1.06, 1.16)		1.02	(0.97, 1.08)	
	Unknown zipcode	0.98	(0.91, 1.06)		1.00	(0.92, 1.08)		0.95	(0.86, 1.04)	

© 2023 Williams SB et al. *JAMA Network Open.*

eTable 6. Odds Ratios for Agent Orange Exposure and Muscle-Invasive Bladder Cancer (MIBC) Stratified by Median Age, Body Mass Index (BMI) Group, Smoking Status, and Charlson Comorbidity Index (CCI) at Bladder Cancer Diagnosis

Variable	Group	OR†	95% CI	p-value for interaction*
Age				0.33
	Above median	0.88	(0.80, 0.97)	
	Below median	0.94	(0.85, 1.04)	
BMI				0.23
	<25	0.95	(0.85, 1.07)	
	25-29.9	0.80	(0.69, 0.92)	
	≥30	0.95	(0.85, 1.06)	
	Unknown	0.90	(0.15, 5.44)	
Smoking status				0.74
	Never Smoked	0.85	(0.72, 1.00)	
	Current Smoker	0.90	(0.77, 1.04)	
	Former Smoker	0.93	(0.84, 1.03)	
	Unknown	0.96	(0.79, 1.18)	
CCI				0.82
	0	0.94	(0.79, 1.12)	
	1	0.84	(0.69, 1.02)	
	2	0.94	(0.78, 1.13)	
	3+	0.91	(0.83, 1.00)	

†Odds ratio for agent orange (yes) among given group

*Corresponds to p-value from interaction term between agent orange and the given variable in multivariable model adjusted for the covariates in Table 4
