# Supplementary material for: Exposure to Agent Orange and Risk of Bladder Cancer Among US Veterans
Source: JAMA Netw Open. 2023 Jun 27;6(6):e2320593. doi: 10.1001/jamanetworkopen.2023.20593 (PMC10300690; doi:10.1001/jamanetworkopen.2023.20593)
Supplement: Supplement 2. — Data Sharing Statement [file jamanetwopen-e2320593-s002.pdf]

Data Sharing Statement

Williams. Exposure to Agent Orange and Risk of Bladder Cancer Among US Veterans. *JAMA Netw Open*. Published June 27, 2023. doi:10.1001/jamanetworkopen.2023.20593

Data
Data available: No
